# Intramuscular injection of human chorionic gonadotropin as luteal phase support in artificial cycle frozen-thawed embryo transfer does not improve clinical outcomes: a parallel, open-label randomized trial

**DOI:** 10.3389/fendo.2023.1283197

**Published:** 2024-01-08

**Authors:** Xiaofang Li, Yu Huang, Zan Shi, Juanzi Shi, Na Li

**Affiliations:** ^1^ Assisted Reproduction Center, Northwest Women’s and Children’s Hospital, Xi’an, Shaanxi, China; ^2^ Department of Reproductive Medicine, Xian Yang Central Hospital, Xianyang, Shaanxi, China

**Keywords:** intramuscular injection of hCG, frozen embryo transfer, ongoing pregnancy rate, artificial cycle, randomized controlled trial

## Abstract

**Background:**

Human chorionic gonadotropin (hCG) as one of the first signals secreted by the embryo to the mother may have a direct effect on the endometrium at implantation. The current study was aim to compare the clinical outcomes after frozen-thawed embryo transfer (FET) treated with artificial cycles (AC) between women who were administered intramuscular injection of human chorionic gonadotropin (hCG) as luteal phase support and the routine group.

**Methods:**

A randomized controlled trial of 245 women was conducted at the Assisted Reproduction Center, Northwest Women’s and Children’s Hospital, Xi’an, China from January 2019 to January 2020. Women <40 years of age undergoing their first FET treated with AC were included. Patients were randomly allocated into either: (1) the hCG treatment group, who received intramuscular injection of hCG since the third day of progesterone administration, at a dose of 2000 IU once every two days, for a total of four times, (2) the control group, receiving routine protocol without placebo on these four days. Clinical outcomes of the two groups were analyzed.

**Results:**

The primary outcome ongoing pregnancy rate in the hCG treatment group versus the control group was 73/124 (58.87%) versus 75/121 (61.98%), respectively (odds ratio [OR], 95% confidence interval [CI]:0.88, 0.53-1.47, *P* = 0.619). Secondary clinical outcomes including biochemical pregnancy, clinical pregnancy, early pregnancy loss, multiple pregnancy, live birth and preterm birth were also comparable between the two groups through the univariate analysis and multivariable regression analysis (*P* > 0.05).

**Conclusion:**

In women undergoing AC-FET, there was no significant difference in the clinical outcomes between the hCG treatment group and the control group. Clinicians should be cautious about adding IM-hCG as luteal phase support to improve the clinical outcome after AC-FET.

**Clinical trial registration:**

http://www.chictr.org.cn/showprojen.aspx?proj=32511, identifier ChiCTR1800020342.

## Introduction

The proportion of frozen embryo transfer (FET) has increased dramatically in recent years. Artificial cycle (AC) is one of the classic schemes for endometrium preparation, owing to its advantages of less monitoring and lower cancellation rate. AC-FET is suitable for women with ovulation disorder, irregular menstruation or those who do not wish to be monitored frequently. During AC-FET, a gonadotropin releasing hormone agonist (GnRH-a) may be used, followed by sequential supplementation with estrogen and progesterone to promote the proliferation and transformation of endometrium (1, 2). A randomized controlled trial (RCT) suggested that estrogen could be tapered from the day a biochemical pregnancy is established without being detrimental to the clinical pregnancy rate in AC-FET cycles (3). Progesterone should be continued until 10-12 weeks of gestation owing to the absence of corpus luteum (4, 5).

Human chorionic gonadotropin (hCG) is one of the first signals secreted by the embryo to the mother (6). HCG is always used in fresh embryo transfer cycles of *in-vitro* fertilization (IVF) or natural cycles-FET for luteal phase support to maintain progesterone secretion by the corpus luteum (7, 8). HCG has been described as one of the modulators of the implantation site by different molecular pathways and through supporting different immune cells (9). The identification of hCG receptors in the endometrium suggested that hCG may have a direct effect on the endometrium at implantation (10, 11). The direct function of hCG on the endometrium to regulate the implantation process may represent a promising direction apart from its traditional function of stimulating corpus luteum. In embryo culture media, hCG is detected from the stage of fertilization (2PN) (12). However, in ART, since embryos are transferred into the uterus at D3 (cleaved embryo) or D5 (blastocyst), the endometrium lacks stimulation from early embryo-derived hCG. Several studies investigated the function of hCG in AC-FET cycles and showed contradictory results, due to limited power with small sample sizes (13–15). Therefore, whether hCG supplementation before the embryo transfer would be beneficial for the implantation of embryos and ultimately improve the clinical outcomes of women after AC-FET remain unclear.

This prospective RCT aimed to compare the clinical outcomes in AC-FET cycles with and without IM-hCG as luteal phase support at a single center.

## Materials and methods

### Study design and study population

This single-center RCT was conducted at the Assisted Reproduction Center, Northwest Women’s and Children’s Hospital, Xi’an, China. The study protocol was approved by the ethics committee of Northwest Women’s and Children’s Hospital (approval number: 2018027), and was registered as ChiCTR1800020342 at http://www.chictr.org.cn/showprojen.aspx?proj=32511. All women participating in the study provided written informed consent. Participants were able to withdraw from the trial at any time.

Participants’ enrollment was scheduled to be completed from January 10, 2019 to January 10, 2020. But the number of planned recruits was not reached by January 10, 2020. We had planned to apply for extending the trial to enroll enough patients, but our center suspended all new IVF treatments due to the COVID-19 pandemic in January 24, 2020. Hence, the trial recruitment was terminated on January 10, 2020.

Women were eligible if they met the following inclusion criteria (1): age < 40 years (2); first FET cycle (3); artificial cycle for endometrium preparation. Women with confirmed endometriosis, uterine malformation, intrauterine adhesion or untreated hydrosalpinx were excluded. Cycles were not eligible if the endometrial thickness was ≤ 8 mm before starting progesterone. Cycles with follicular diameter >14 mm on the day of progesterone administration were also excluded. Women were also excluded if they were participating in other studies.

### Randomization

We selected women who met the inclusion criteria and started daily endometrial preparation for FET. The details of the trial were explained to the women by a member of the project, and eligible women who signed the consent form were randomized on the day of progesterone administration. Women were randomly allocated to the hCG treatment or the control group according to a randomization list generated by a computer. The specific process was as follows: 300 random numbers were produced by a computer and divided into A and B groups, with 150 in each group. Then a random group table was made and blinded on computer. The 300 random numbers obtained above were randomly distributed to 300 sequence numbers. For every patient included, a random number was obtained according to the order of inclusion. Then the number was unblinded by the computer.

The randomization process was completed by a member of the project. Therefore, the staff who conduct the randomization process and the participants were not blinded. The physician who performed the endometrial preparation protocol and determined the number and grade of embryos transferred was blinded to the grouping. Laboratory staff and staff who conducted the data analysis and follow-up were blinded to the allocation.

### Preparation of endometrium and luteal phase support

Women received a transvaginal ultrasound on the fifth day of menstruation if their urine hCG examination was negative. Estrogen was administered at starting doses of 4-6 mg/d for five days (oral estradiol valerate tablets, Bayer, Germany), and then adjusted after evaluating the endometrial growth by transvaginal ultrasound. Intramuscular (IM) progesterone (Zhejiang Xianju Pharmaceutical Co., Ltd.) was commenced if the endometrium was ≥ 8 mm and serum progesterone value was < 1.5 ng/ml. The transfer of cleaved embryo or blastocyst was performed after 4 days (20mg per day for one day, then 40mg per day for 2 days, then 60mg per day for 1 day) or 6 days (20mg per day for one day, then 40mg per day for 2 days, then 60mg per day for 3 days) of progesterone administration. For the gonadotropin-releasing hormone (GnRH-a)-AC cycles, GnRH-a (3.75 mg, Beaufort, France) was administered on the 2-4 day of menstruation. Estrogen was started approximately 30 days later as described above for the AC-FET scheme.

**Figure 1:**
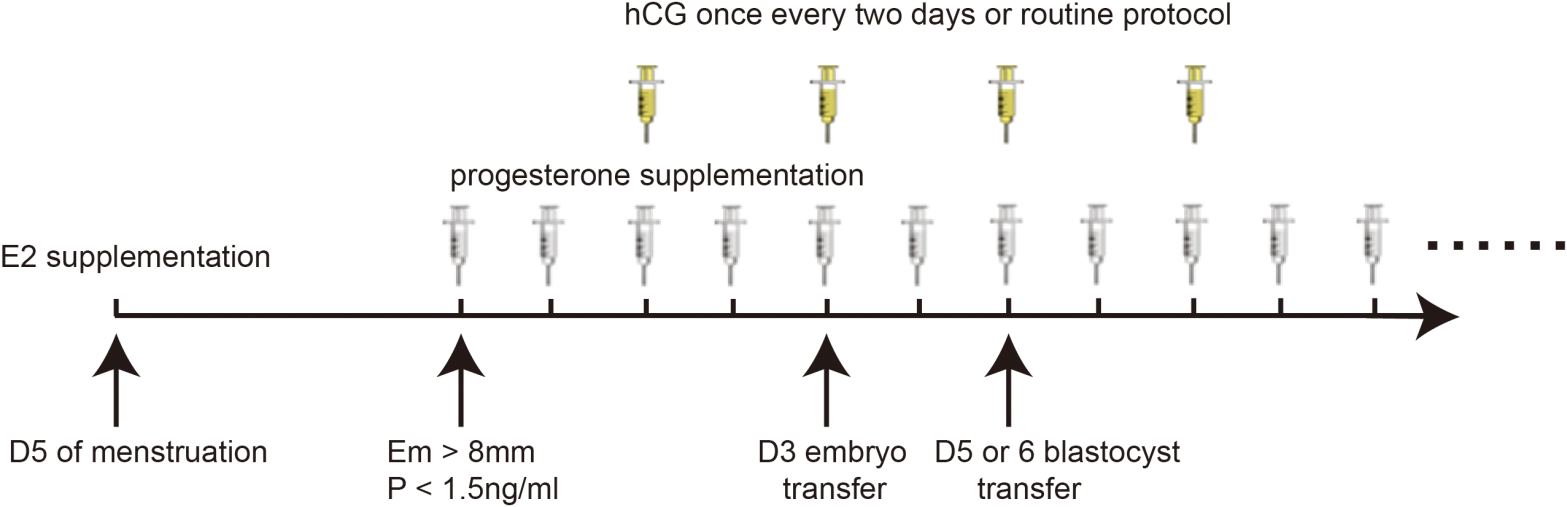
Specific programmes for the study.

In the hCG treatment group, women received IM injection of hCG since the third day of starting progesterone, at a dose of 2000 IU once every two days, for a total of four times. The control group received routine protocol without placebo on these four days (**Figure 1**).

In addition to continuing progesterone (60mg daily) and estrogen, 20 mg of dydrogesterone (Duphaston, Abbott Biologicals B.V.) was added daily until 10 weeks of gestation. The dose of estrogen was tapered on the 12th or 14th day after cleaved embryo or blastocyst transfer if biochemical pregnancy was confirmed. The dose of progesterone was tapered every three days from the 10th gestational week.

### Outcomes

The primary outcome of the present study was ongoing pregnancy, defined as the process of pregnancy beyond 12th week of gestation. The secondary outcomes included biochemical pregnancy: hCG test was positive after 12 (blastocyst) or 14 (cleaved embryo) days of transfer, clinical pregnancy (CP): the presence of intrauterine sac on ultrasound at six weeks of gestation, early pregnancy loss: spontaneous miscarriage before 12 weeks of pregnancy or no gestational sac was confirmed after biochemical pregnancy, multiple pregnancy: more than one gestational sac or embryo bud detected on ultrasound at 6-8 weeks of gestation and preterm birth: a baby born alive at 24-37 weeks of gestational age. At the present stage, we have already obtained the live birth data (defined as the delivery of a live baby at more than 24 weeks of gestation). As the live birth rate is the most concerned outcome for both patients and physicians, we also reported the live birth outcome as one of the secondary outcomes.

All participants received allocated intervention and their follow-up data were all acquired in the present study.

### Sample size calculation

A superiority design was performed as hCG may have a direct function on the endometrium at implantation and result in a higher ongoing pregnancy rate than the routine group. According to Maryam Eftekhar et al., clinical pregnancy rate was 16% in the control group, and 28% in women who received three doses of hCG after embryo transfer (13). To demonstrate 12% increase of ongoing pregnancy rate with a one-sided test, 80% power, and 0.05 alpha error, 147 women at least were required in each group.

### Statistical analysis

The analyses in the current study were conducted according to the intention-to-treat principle. The normality of continuous variables was tested by Kolmogorov–Smirnov test. For non-normally distributed continuous variables, the means (25th-75th percentiles) were displayed. Categorical parameters were presented as frequencies (percentages). Kruskal-Wallis test for continuous variables and Pearson’s χ2 test or Fisher’s exact test for qualitative data were performed, where appropriate. Univariate analysis was performed to examine the relationship between hCG treatment and clinical outcomes. Multivariate logistic regression analysis was conducted to adjust potential confounders and to further identify the association of hCG treatment with clinical outcomes in AC-FET cycles. Interaction and stratified analyses were performed according to protocol in the FET cycle (AC and GnRH-a+AC), type of embryo transferred (cleavage stage and blastocyst stage), number of embryos transferred (1 and 2) and number of good quality embryos transferred (0,1 and 2). A *P* value < 0.05 was considered statistically significant. All analyses were performed using IBM® SPSS® software (version: 22.0, SPSS Inc. Headquarters, USA), the statistical packages R (The R Foundation; http://www.r-project.org; version 3.4.3) and EmpowerStats (www.empowerstats.com; version: 3.0, X&Y Solutions Inc.).

## Results

### Demographic and clinical data of the fresh IVF cycles

**Figure 2:**
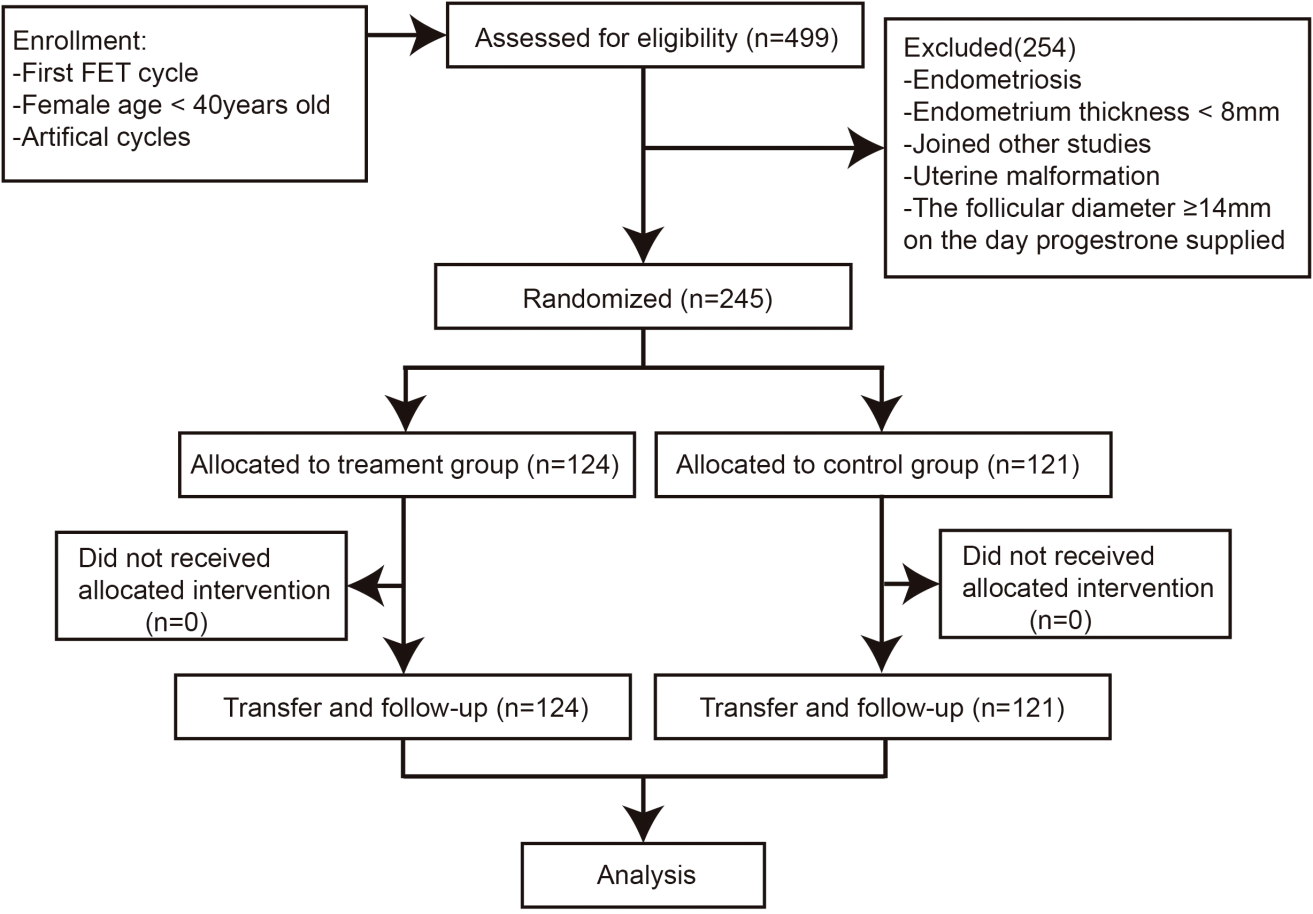
Flowchart of the study cohort.

Between January 2019 and January 2020, a total of 245 women were eligible and randomly allocated to the hCG treatment group (n = 124) or the control group (n = 121) (**Figure 2**). Although women in the hCG treatment group had less tubal factors causing infertility when compared with women in the control group (35.48% versus 43.80%, *P* > 0.05), there were no significant differences between the two groups in the baseline characteristics and clinical data of the fresh IVF cycles (**Table 1**).

**Table 1:** Demographic and clinical data of the fresh IVF cycles.

Variables	Control group	Treatment group	*P*-value
n	121	124	
Women’s age at oocyte retrieval (years)	29.17 (26–31)	29.94 (28–32)	0.063^KW^
BMI (kg/m2)	23.02 (19.83-25.39)	23.05 (20.16-25.39)	0.824^KW^
Infertile years	3.60 (2–5)	3.86 (2–5)	0.503^KW^
Ovarian reserve function			
bFSH (mIU/mL)	6.92 (5.66-7.71)	6.49 (5.36-7.54)	0.189^KW^
AFC	15.23 (10–22)	14.21 (9–20)	0.201^KW^
Cause of infertility			0.757^K^
Tubal factor	53 (43.80%)	44 (35.48%)	
Ovulation disorder	9 (7.44%)	8 (6.45%)	
Diminished ovarian reserve	2 (1.65%)	2 (1.61%)	
Male factor	20 (16.53%)	21 (16.94%)	
Unexplained	10 (8.26%)	12 (9.68%)	
More than one etiology	27 (22.31%)	37 (29.84%)	
First IVF cycle	116 (95.87%)	112 (90.32%)	0.088^K^
Protocol in fresh cycle			0.385^K^
Agonist	94 (77.69%)	92 (74.19%)	
Antagonist	24 (19.83%)	31 (25.00%)	
Others	3 (2.48%)	1 (0.81%)	
Gonadotropin dosage (IU)	2101.24 (1500–2525)	2299.80 (1500–3000)	0.577^KW^
Duration of stimulations (days)	10.96 (9–12)	11.55 (9–13)	0.249^KW^
Oestradiol level on the hCG day (pg/mL)	5500.62 (2803.00-7206.00)	5213.74 (3109.25-6615.75)	0.796^KW^
Progesterone level on the hCG day (ng/mL)	1.60 ± 0.81	1.42 ± 0.66	0.116^KW^
Insemination			0.195^K^
IVF	99 (81.82%)	93 (75.00%)	
ICSI	22 (18.18%)	31 (25.00%)	
Number of oocytes retrieved	13.92 (10–18)	13.83 (9–17)	0.807^KW^
Total number of good quality embryo available	5.24 (3–7)	4.98 (2–7)	0.483^KW^

KW, Kruskal–Wallis test; K, Pearson’s chi-square test; BMI, body mass index; AFC, antral follicle count; FSH, follicle-stimulating hormone; IVF, in-vitro fertilization; hCG, human chorionic gonadotropin; ICSI, Intracytoplasmic sperm injection; Data presented as number (percentage) or median (25th–75th centiles). Both groups were found no statistical significance.

In terms of the characteristics of FET cycles, there were more blastocyst stage embryos transferred and more single embryo transfer cycles in the control group as compared to the treatment group (88.43% versus 76.61% and 86.78% versus 74.19%, *P* = 0.015 and 0.013, respectively). Women in the control group were younger at embryo transfer when compared with women in the hCG treatment group (*P* = 0.046). Other parameters including the protocol used in FET cycles (pretreatment with GnRH-a or not), endometrial thickness on the day of progesterone administration, triple-line endometrial pattern and number of good quality embryos transferred were all similar between the two groups (*P* > 0.05) (**Table 2**).

**Table 2:** Characteristics of FET cycles.

Variables	Control group	Treatment group	*P*-value
n	121	124	
Women’s age at embryo transfer (years)	29.46 (27–32)	30.4 (28–33)	**0.046^KW^**
FET protocol			0.406^K^
AC	84 (69.42%)	92 (74.19%)	
GnRH-a+AC	37 (30.58%)	32 (25.81%)	
Em (mm)	10.6 (9.5-11.6)	10.4 (9.5-11.0)	0.322^KW^
Triple-line endometrial pattern			0.775^K^
A	34 (28.10%)	40 (32.26%)	
B	75 (61.98%)	72 (58.06%)	
C	12 (9.92%)	12 (9.68%)	
Type of embryo transferred			**0.015^K^**
Cleavage stage	14 (11.57%)	29 (23.39%)	
Blastocyst stage	107 (88.43%)	95 (76.61%)	
Number of embryos transferred			**0.013^K^**
1	105 (86.78%)	92 (74.19%)	
2	16 (13.22%)	32 (25.81%)	
Number of good quality embryos transferred			0.082^KW^
0	24 (19.83%)	40 (32.26%)	
1	88 (72.73%)	75 (60.48%)	
2	9 (7.44%)	9 (7.26%)	

Significant difference values are in bold.

AC, artificial cycle; GnRH-a, gonadotropin-releasing hormone-agonist;

Em, endometrial thickness on the day progesterone commenced.

### Clinical outcomes

The primary outcome, ongoing pregnancy rate in the treatment group versus the control group was 58.87% versus 61.98% (OR: 0.88, 95% CI: 0.53-1.47). No differences were found between the two groups in terms of biochemical pregnancy rate (75.81% versus 76.03%, OR: 0.99, 95% CI: 0.55-1.77), implantation rate (57.69% versus 68.61%, OR: 0.62, 95% CI: 0.39-1.01), clinical pregnancy rate (66.13% versus 73.55%, OR: 0.70, 95% CI: 0.41-1.22), early pregnancy loss rate (22.34% versus 18.48%, OR: 1.12, 95% CI: 0.82-1.56), multiple pregnancy rate (7.23% versus 6.61%, OR: 1.05, 95% CI: 0.66-1.67), live birth rate (55.65% versus 57.85%, OR: 0.91, 95% CI: 0.55-1.52) and preterm birth rate (20.29% versus 14.29%, OR: 1.22, 95% CI: 0.83-1.80) (treatment group versus control group, respectively). No ectopic pregnancy occurred in the two groups (**Table 3**).

**Table 3:** Clinical outcomes.

Variables	Control group	Treatment group	OR (95% CI)	*P*-value
n	121	124		
Primary outcome				
Ongoing pregnancy rate (%)	75/121 (61.98%)	73/124 (58.87%)	0.88 (0.53,1.47)	0.619
Secondary outcomes				
Biochemical pregnancy rate (%)	92/121 (76.03%)	94/124 (75.81%)	0.99 (0.55, 1.77)	0.967
Implantation rate (%)	94/137 (68.61%)	90/156 (57.69%)	0.62 (0.39, 1.01)	0.054
Clinical pregnancy rate (%)	89/121 (73.55%)	82/124 (66.13%)	0.70 (0.41, 1.22)	0.207
Early pregnancy loss rate (%)	17/92 (18.48%)	21/94 (22.34%)	1.12 (0.81, 1.56)	0.514
Multiple pregnancy a rate (%)	8/121 (6.61%)	9/124 (7.23%)	1.05 (0.66, 1.67)	0.842
Ectopic pregnancy	0	0		
Live birth rate (%)	70/121 (57.85%)	69/124 (55.65%)	0.91 (0.55, 1.52)	0.728
Preterm birth rate (%)	10/70 (14.29%)	14/69 (20.29%)	1.22 (0.83, 1.80)	0.349

a All multiple pregnancies in both groups were twin pregnancies.

OR, odds ratio; CI, confidence interval.

### Association of hCG treatment with clinical outcomes in AC-FET cycles

The multivariate logistic regression models were conducted to assess the association between hCG treatment in AC-FET cycles and clinical outcomes, while adjusting for potential confounding factors. In the adjusted model 1 and adjusted model 2, hCG treatment was not a significant factor for ongoing pregnancy (model1:OR: 0.84, 95% CI: 0.48-1.47 and model2:OR: 1.01, 95% CI: 0.53-1.94), live birth (model1:OR: 0.85, 95% CI: 0.49-1.49 and model2:OR: 1.04, 95% CI: 0.54-2.00), biochemical pregnancy (model1:OR: 0.99, 95% CI: 0.52-1.86 and model2:OR: 1.12, 95% CI: 0.54-2.33), clinical pregnancy (model1:OR: 0.67, 95% CI: 0.37-1.22 and model2:OR: 0.70, 95% CI: 0.35-1.40), implantation (model1:OR: 0.61, 95% CI: 0.36-1.05 and model2:OR: 0.84, 95% CI: 0.47-1.52) and early pregnancy loss (model1:OR: 1.61, 95% CI: 0.71-3.63 and model2:OR: 1.36, 95% CI: 0.54-3.40) (**Table 4**).

**Table 4:** Logistic regression analysis of the clinical outcomes.

	Control group	Treatment group
n	121	124
Primary outcome		
Ongoing pregnancy rate		
Adjusted model 1 OR (95%CI)	Reference	0.84 (0.48, 1.47)
Adjusted model 2 OR (95%CI)	Reference	1.01 (0.53, 1.94)
Secondary outcomes		
Biochemical pregnancy		
Adjusted model 1 OR (95%CI)	Reference	0.99 (0.52, 1.86)
Adjusted model 2 OR (95%CI)	Reference	1.12 (0.54, 2.33)
Clinical pregnancy		
Adjusted model 1 OR (95%CI)	Reference	0.67 (0.37, 1.22)
Adjusted model 2 OR (95%CI)	Reference	0.70 (0.35, 1.40)
Implantation rate		
Adjusted model 1 OR (95%CI)	Reference	0.61 (0.36, 1.05)
Adjusted model 2 OR (95%CI)	Reference	0.84 (0.47, 1.52)
Early pregnancy loss		
Adjusted model 1 OR (95%CI)	Reference	1.61 (0.71, 3.63)
Adjusted model 2 OR (95%CI)	Reference	1.36 (0.54, 3.40)
Live birth		
Adjusted model 1 OR (95%CI)	Reference	0.85 (0.49, 1.49)
Adjusted model 2 OR (95%CI)	Reference	1.04 (0.54, 2.00)

OR, odds ratio; CI, confidence interval.

Adjusted Model I: we adjusted for women’s age at retrieval; BMI and infertile years.

Adjusted Model II: we adjusted for women’s age at retrieval and embryo transfer; BMI; infertile years; bFSH; AFC; protocol in fresh cycle; number of oocytes retrieved; fertilization type; Em; triple-line endometrial pattern; type of embryo transferred; number of transferred embryos and number of good-quality embryos transferred.

### Subgroup analyses

Further subgroup analyses were conducted based on FET protocol, type of embryo transferred (cleavage and blastocyst stage), number of embryos transferred (1 and 2) and number of good quality embryos transferred (0,1 and 2) to assess the stability of association of hCG administration in AC-FET cycles and clinical outcomes. The results demonstrated that no significant differences were observed on ongoing pregnancy, live birth, clinical pregnancy and early pregnancy loss between hCG treatment and control group, in all subgroups and no significant interactions were found in any of the subgroups (*P* > 0.05 for all comparisons) (**Supplementary Figures 1**–**4**).

## Discussion

The present study found no significant differences in ongoing pregnancy and live birth rates between additional hCG supplementation as luteal phase support before the embryo transfer compared with the routine protocol in women undergoing AC-FET.

Traditionally, hCG plays an essential role in maintaining progesterone secretion by corpus luteum in the early stage of pregnancy and it is also used in natural or stimulated cycles in FET to induce ovulation by mimicking the LH surge. However, the transcription of hCG gene by embryo begins very early due the micro-amount of hCG has been detected from the stage of 2PN in embryo culture medium. With the identification of endometrial hCG receptors, it has been thus proposed that hCG may have direct effects on embryo-endometrial communication during implantation of human embryos (11). The application of hCG improves endometrial receptivity by inhibiting the expression of endometrial insulin-like growth factor binding protein-1 (16, 17), while increasing the expression of homeobox A10 (18). Moreover, hCG may stimulate angiogenesis during implantation by targeting VEGF/MEK/ERK or VEGF/NF-κB signaling pathway (19–21). HCG also plays a paracrine role by stimulating the leukemia inhibitory factor or inhibiting macrophage colony stimulating factor, which are important cytokines during implantation (22). On the basis of foregoing, in AC-FET cycles, hCG is likely an adjuvant therapy to enhance the clinical outcome in addition to the essential estrogen and progesterone.

Intrauterine administration of hCG has been suggested to improve clinical outcome in IVF patients. Many studies have investigated the effect of intrauterine hCG infusion on clinical outcomes but the conclusions of these studies were inconsistent due to the heterogeneity of study design (23–25). Mansour et al. reported that pregnancy rate was significantly increased by intrauterine hCG infusion which was in line with Zarei et al’s conclusion (26, 27). Two recent RCTs conducted by Barbara Wirleitner et al. and Karim S. Abdallah et al. suggested that intrauterine hCG supplementation does not increases pregnancy rates in IVF patients (23, 24).

The endometrial cells were exposed to exogenous hCG for only a short time when infusion of hCG. However, in experiments conducted by Sherwin et al. showed that prolonged exposure of hCG may be down regulate the pro-implantation factors and have deleterious effects on endometrial receptivity (28). Therefore, moderate hCG supplementation may be more beneficial to clinical outcomes. Asgerally T. Fazleabas et al. suggested that continuous presence of hCG was needed to sustain the impact of the initial single-dose intrauterine hCG (29), indicating that multiple hCG administrations may provide benefits. Intrauterine perfusion after the embryo transfer is obviously not feasible. However, IM-HCG can be given both before and after the embryo transfer. Therefore, the effects of IM-hCG for women undergoing AC-FET were investigated.

There is no consensus on the optimal schedule, such as dose, time point and frequency of supplementation for the introduction of hCG. Some schemes have been reported, including 3000 IU every three days since the third day of starting progesterone for three doses (14), 5000 IU every three days for three doses after embryo transfer (13), 250 μg of recombinant hCG every three days from the day of progesterone initiation for three doses (15). As the half time of hCG is 24-36 hours (30), we used 2000 IU once every two days since the third day of progesterone initiation, for a total of four times, which was somewhat center-selective. However, there was no significant difference between the hCG treatment group and the control group in terms of clinical pregnancy rate, which was in accordance with some previous studies (14, 15), but in contrast to the study of Afsar et al. (13). However, there was a marginal difference with a small sample size (Chemical pregnancy rate: *P* = 0.048 between the two groups) in the study of Afsar et al. and they did not conduct multivariate regression analysis to control confounding factors (13–15). In addition, ongoing pregnancy and live birth outcomes were not reported in these studies. In the present study, all live births were followed-up and no difference was found between the two groups. Larger sample size and multi-center studies are needed to confirm these findings.

The main strength of the current study is the sufficient follow-up data to report ongoing pregnancy and live birth rate, which is the most important outcome for patients undergoing IVF. The complete follow-up for all women was another strength of the present study. Besides, we conducted logistic regression analysis and subgroup analysis to ensure the stability of the conclusion. On the other hand, our study only included the first FET cycle, which may minimize some potential bias from patients and clinicians (such as clinic variability or patients’ psychological factor). Furthermore, in order to reduce bias, the physician who conducted endometrial preparation protocol and staff who conducted the follow-up were blinded to the group assignment.

This study had some limitations. First, some characteristics of FET cycles, such as type and number of embryos transferred, differed between the groups. However, multivariable logistic analysis and subgroup analysis were performed to minimize the potential impact. Second, as this was a clinical trial, it could not explain the mechanism of the ineffectiveness of IM-hCG on clinical outcome. It may be due to the insufficient concentration of hCG in the endometrium. However, at least, the schedule we used had no significant difference when compared with the control group in terms of clinical outcomes. Third, the COVID-19 pandemic resulted in lockdown from January 24, 2020, and all IVF treatments were halted at our hospital. More than 80% (245/294) of the calculated sample size was reached at that time. In addition, it is difficult to require women to visit the clinic or hospital for an HCG injection every day. We stopped our trial early even though the lockdown was lifted two months later. Nevertheless, all the follow-up data were complete. The current study provided the ongoing pregnancy rates of the two groups based on the admittance standards during the study period. Lastly, no significant difference was found in terms of ongoing pregnancy and live birth rate in women undergoing AC-FET with or without additional hCG administration as luteal phase support through the multivariate analysis and subgroup analysis. The data could be a reference for the multi-center with larger sample sizes analyses and molecular mechanism research. And the trial could also be useful to include in a meta-analysis with other available evidence in the future.

In conclusion, clinicians should be cautious in recommending IM-hCG as an adjuvant therapy to improve clinical outcomes, and the addition of hCG may impose unnecessary financial burden on patients. More multi-center, larger sample sizes analyses are needed to validate the results of this study. In addition, further studies on the physiological level are also needed to analyze the molecular mechanisms of the effect of hCG on embryonic-maternal cross-talk.

## Data availability statement

The raw data supporting the conclusions of this article will be made available by the authors, without undue reservation.

## Ethics statement

The studies involving humans were approved by Ethics committee of the Northwest Women’s and Children’s Hospital (number: 2018027). The studies were conducted in accordance with the local legislation and institutional requirements. The participants provided their written informed consent to participate in this study.

## Author contributions

XL: Data curation, Formal Analysis, Investigation, Methodology, Project administration, Software, Validation, Visualization, Writing – original draft, Writing – review & editing. YH: Data curation, Investigation, Methodology, Resources, Writing – review & editing. ZS: Data curation, Investigation, Methodology, Resources, Writing – review & editing. JS: Conceptualization, Project administration, Writing – review & editing. NL: Conceptualization, Formal Analysis, Funding acquisition, Investigation, Methodology, Project administration, Writing – review & editing.

## References

[1] Salemi S Yahyaei A Vesali S Ghaffari F. Endometrial preparation for vitrified-warmed embryo transfer with or without gnrh-agonist pre-treatment in patients with polycystic ovary syndrome: A randomized controlled trial. Reprod BioMed Online (2021) 43(3):446–52. doi: 10.1016/j.rbmo.2021.06.006 34340936

[2] Azimi Nekoo E Chamani M Shahrokh Tehrani E Hossein Rashidi B Davari Tanha F Kalantari V. Artificial endometrial preparation for frozen-thawed embryo transfer with or without pretreatment with depot gonadotropin releasing hormone agonist in women with regular menses. J Family Reprod Health (2015) 9(1):1–4. 25904960 PMC4405510

[3] Zhao J Huang X Zeng Q Sun L Liu N Li Y. Oestrogen dose tapering during luteal phase does not affect clinical outcomes after hormone replacement treatment-frozen-thawed embryo transfer cycles: A retrospective analysis. Hum Reprod (2019) 34(8):1479–84. doi: 10.1093/humrep/dez096 31310320

[4] Nakajima ST Nason FG Badger GJ Gibson M. Progesterone production in early pregnancy. Fertil Steril (1991) 55(3):516–21. 2001753

[5] Bourdon M Santulli P Kefelian F Vienet-Legue L Maignien C Pocate-Cheriet K. Prolonged estrogen (E2) treatment prior to frozen-blastocyst transfer decreases the live birth rate. Hum Reprod (Oxford England) (2018) 33(5):905–13. doi: 10.1093/humrep/dey041 29529202

[6] Schumacher A Zenclussen AC. Human chorionic gonadotropin-mediated immune responses that facilitate embryo implantation and placentation. Front Immunol (2019) 10:2896. doi: 10.3389/fimmu.2019.02896 31921157 PMC6914810

[7] Strott CA Yoshimi T Ross GT Lipsett MB. Ovarian physiology - relationship between plasma lh and steroidogenesis by follicle and corpus luteum - effect of Hcg. J Clin Endocrinol Metab (1969) 29(9):1157–+. doi: 10.1210/jcem-29-9-1157 5808525

[8] Haas J Lantsberg D Feldman N Manela D Orvieto R. Modifying the luteal phase support in natural cycle frozen-thawed embryo transfer improves cycle outcome. Gynecol Endocrinol (2015) 31(11):891–3. 10.3109/09513590.2015.1075502 26288149

[9] Makrigiannakis A Vrekoussis T Zoumakis E Kalantaridou SN Jeschke U. The role of hcg in implantation: A mini-review of molecular and clinical evidence. Int J Mol Sci (2017) 18(6):1305. doi: 10.3390/ijms18061305 28629172 PMC5486126

[10] Licht P von Wolff M Berkholz A Wildt L. Evidence for cycle-dependent expression of full-length human chorionic gonadotropin/luteinizing hormone receptor mrna in human endometrium and decidua. Fertil Steril (2003) 79:718–23. doi: 10.1016/s0015-0282(02)04822-7 12620482

[11] Licht P Fluhr H Neuwinger J Wallwiener D Wildt L. Is human chorionic gonadotropin directly involved in the regulation of human implantation? Mol Cell Endocrinol (2007) 269(1-2):85–92. doi: 10.1016/j.mce.2006.09.016 17367920

[12] Chen XY Li J Jiang D Li T Liu XR Zhuang GL. A highly sensitive electrochemiluminescence immunoassay for detecting human embryonic human chorionic gonadotropin in spent embryo culture media during Ivf-Et cycle. J Assist Reprod Genet (2013) 30(3):377–82. doi: 10.1007/s10815-012-9923-7 PMC3607682 23274513

[13] Eftekhar M Dashti S Omidi M Tabatabaei AA. Does luteal phase support by human chorionic gonadotropin improve pregnancy outcomes in frozen-thawed embryo transfer cycles? Middle East Fertil Soc J (2018) 23(4):300–2. doi: 10.1016/j.mefs.2018.03.003

[14] Shiotani M Matsumoto Y Okamoto E Yamada S Mizusawa Y Furuhashi K. Is human chorionic gonadotropin supplementation beneficial for frozen and thawed embryo transfer in estrogen/progesterone replacement cycles?: A randomized clinical trial. Reprod Med Biol (2017) 16(2):166–9. 29259465 10.1002/rmb2.12023 PMC5661815

[15] Ben-Meir A Aboo-Dia M Revel A Eizenman E Laufer N Simon A. The benefit of human chorionic gonadotropin supplementation throughout the secretory phase of frozen-thawed embryo transfer cycles. Fertil Steril (2010) 93(2):351–4. 10.1016/j.fertnstert.2009.02.027 19342020

[16] Licht P Russu V Lehmeyer S,M Ll J Siebzehnrübl E Wildt L. Intrauterine microdialysis reveals cycle-dependent regulation of endometrial insulin-like growth factor binding protein-1 secretion by human chorionic gonadotropin. Fertil Steril (2002) 78(2):252–8. 10.1016/s0015-0282(02)03226-0 12137859

[17] Fluhr H Carli S Deperschmidt M Wallwiener D Zygmunt M Licht P. Differential effects of human chorionic gonadotropin and decidualization on insulin-like growth factors-I and -ii in human endometrial stromal cells. Fertil Steril (2008) 90(4-supp-S):1384–9. 10.1016/j.fertnstert.2007.07.1357 18022169

[18] Zhu M Yi S Huang X Meng J Sun H Zhou J. Human chorionic gonadotropin improves endometrial receptivity by increasing the expression of homeobox A10. Mol Hum Reprod (2020) 6):6. 10.1093/molehr/gaaa026 32502249

[19] Jing G Yao J Dang Y Liang W Li Z. The role of B-Hcg and Vegf-Mek/Erk signaling pathway in villi angiogenesis in patients with missed abortion. Placenta (2021) 103(7):16–23. 33068962 10.1016/j.placenta.2020.10.005

[20] Zhang Z Huang Y Zhang J Liu Z Lin Q Wang Z. Activation of nf-Kb signaling pathway during hcg-induced vegf expression in luteal cells. Cell Biol Int (2018) 43(3):344–9. 10.1002/cbin.11090 30597662

[21] Berndt S D”Hauterive SP Blacher S Pequeux C Lorquet S Munaut C. Angiogenic activity of human chorionic gonadotropin through lh receptor activation on endothelial and epithelial cells of the endometrium. FASEB J (2006) 20(14):2630–2. 10.1096/fj.06-5885fje 17065221

[22] Perrier d Charlet-Renard C Berndt S Dubois M Munaut C Goffin F. Human chorionic gonadotropin and growth factors at the embryonic-endometrial interface control leukemia inhibitory factor (Lif) and interleukin 6 (Il-6) secretion by human endometrial epithelium. Hum Reprod (2004) 19(11):2633–43. 10.1093/humrep/deh450 15388676

[23] Wirleitner B Schuff M Vanderzwalmen P Stecher A Okhowat J Hradecký L. Intrauterine administration of human chorionic gonadotropin does not improve pregnancy and life birth rates independently of blastocyst quality: A randomised prospective study. Reprod Biol Endocrinol (2015) 13:70. 26141379 10.1186/s12958-015-0069-1 PMC4491277

[24] Abdallah K Makhlouf A Badran E El-Nashar I Al-Hussaini T Farghaly T. Intrauterine injection of hcg before embryo transfer: A parallel, double-blind randomized trial. Reprod BioMed Online (2021) 43(4):663–9. 10.1016/j.rbmo.2021.06.011 34412973

[25] Laokirkkiat P Thanaboonyawat I Boonsuk S Petyim S Prechapanich J Choavaratana R. Increased Implantation Rate after Intrauterine Infusion of a Small Volume of Human Chorionic Gonadotropin at the Time of Embryo Transfer: A Randomized, Double-Blind Controlled Study. Arch Gynecol Obstet (2019) 299(1):267–75. doi: 10.1007/s00404-018-4962-7 30449012

[26] Mansour R Tawab N Kamal O El-Faissal Y Serour A Aboulghar M. Intrauterine injection of human chorionic gonadotropin before embryo transfer significantly improves the implantation and pregnancy rates in invitro fertilization/intracytoplasmic sperm injection: A prospective randomized study. Fertil Steril (2011) 96(6):1370–4.e1. 22047664 10.1016/j.fertnstert.2011.09.044

[27] Zarei A Parsanezhad ME Younesi M Alborzi S Zolghadri J Samsami A. Intrauterine administration of recombinant human chorionic gonadotropin before embryo transfer on outcome of in vitro fertilization/intracytoplasmic sperm injection: A randomized clinical trial. Iranian J Reprod Med (2014) 12(1):1–6. PMC4009584 24799855

[28] Sherwin JRA Sharkey AM Cameo P Mavrogianis PM Catalano RD Edassery S. Identification of novel genes regulated by chorionic gonadotropin in baboon endometrium during the window of implantation. Endocrinology (2007) 2):618–26. 10.1210/en.2006-0832 17110430

[29] Strug MR Renwei S Young JE Dodds WG Shavell VI Patricia D-G. Intrauterine human chorionic gonadotropin infusion in oocyte donors promotes endometrial synchrony and induction of early decidual markers for stromal survival: A randomized clinical trial. Hum Reprod (2016) 7):1552. 10.1093/humrep/dew080 PMC4901879 27122490

[30] d’Hauterive SP Close R Gridelet V Mawet M Nisolle M Geenen V. Human chorionic gonadotropin and early embryogenesis: review. Int J Mol Sci (2022) 23(3):1380. doi: 10.3390/ijms23031380 35163303 PMC8835849

