# Supplementary material for: Intramuscular injection of human chorionic gonadotropin as luteal phase support in artificial cycle frozen-thawed embryo transfer does not improve clinical outcomes: a parallel, open-label randomized trial
Source: Front Endocrinol (Lausanne). 2024 Jan 8;14:1283197. doi: 10.3389/fendo.2023.1283197 (PMC10801214; doi:10.3389/fendo.2023.1283197)
Supplement: Supplementary file 1 [file Table_1.docx]

**Supplemental Table 1 Effect of** **HCG administrated in AC-FET cycles on ongoing pregnancy rate in each subgroup.**

|  | No.of patients | OR (95%CI)^a^ | *P* value | *P* for interaction |
| --- | --- | --- | --- | --- |
| FET protocol |  |  |  | 0.632 |
| AC | 176 | 0.79 (0.41,1.52) | 0.481 |  |
| GnRH-a+AC | 69 | 0.84 (0.24,3.02) | 0.794 |  |
| Type of embryo transferred |  |  |  | 0.749 |
| Cleavage stage | 43 | 0.64(0.10,4.08) | 0.639 |  |
| Blastocyst stage | 202 | 0.90 (0.49,1.65) | 0.723 |  |
| Number of embryos transferred |  |  |  | 0.488 |
| 1 | 197 | 0.82 (0.45,1.51) | 0.530 |  |
| 2 | 48 | 0.20 (0.02,1.68) | 0.137 |  |
| Number of good quality embryos transferred |  |  |  | 0.959 |
| 0 | 64 | 1.00(0.23,4.26) | 1.000 |  |
| 1 | 163 | 0.85 (0.43,1.70) | 0.649 |  |
| 2 | 18 | 0.00 (0.00,inf) | 0.999 |  |

Note: CI = confidence interval; OR = odds ratio; ^a^Adjusted for women’s age at embryo transfer.

**Supplemental Table 2** **Effect of HCG administrated in AC-FET cycles on live birth rate in each subgroup.**

|  | No.of patients | OR (95%CI)^a^ | *P* value | *P* for interaction |
| --- | --- | --- | --- | --- |
| FET protocol |  |  |  | 0.944 |
| AC | 176 | 0.94 (0.51,1.71) | 0.833 |  |
| GnRH-a+AC | 69 | 1.14 (0.42,3.13) | 0.799 |  |
| Type of embryo transferred |  |  |  | 0.529 |
| Cleavage stage | 43 | 0.62 (0.16,2.34) | 0.479 |  |
| Blastocyst stage | 202 | 1.12 (0.64,1.98) | 0.691 |  |
| Number of embryos transferred |  |  |  | 0.305 |
| 1 | 197 | 1.01 (0.57,1.78) | 0.976 |  |
| 2 | 48 | 0.50 (0.12,1.96) | 0.317 |  |
| Number of good quality embryos transferred |  |  |  | 0.941 |
| 0 | 64 | 1.29(0.44,3.80) | 0.6420 |  |
| 1 | 163 | 1.06 (0.57,2.01) | 0.8460 |  |
| 2 | 18 | 0.29 (0.02,5.49) | 0.4121 |  |

Note: CI = confidence interval; OR = odds ratio; ^a^Adjusted for women’s age at embryo transfer.

**Supplemental Table 3 Effect of HCG administrated in AC-FET cycles on clinical pregnancy rate in each subgroup.**

|  | No.of patients | OR (95%CI)^a^ | *P* value | *P* for interaction |
| --- | --- | --- | --- | --- |
| FET protocol |  |  |  | 0.533 |
| AC | 176 | 0.78 (0.41,1.47) | 0.434 |  |
| GnRH-a+AC | 69 | 0.61 (0.19,1.94) | 0.405 |  |
| Type of embryo transferred |  |  |  | 0.775 |
| Cleavage stage | 43 | 0.74 (0.19,2.85) | 0.665 |  |
| Blastocyst stage | 202 | 0.77 (0.42,1.44) | 0.418 |  |
| Number of embryos transferred |  |  |  |  |
| 1 | 197 | 0.83 (0.45,1.52) | 0.544 | 0.055 |
| 2 | 48 | 0.12 (0.01,1.08) | 0.059 |  |
| Number of good quality embryos transferred |  |  |  | 0.551 |
| 0 | 64 | 0.94(0.33,2.73) | 0.916 |  |
| 1 | 163 | 0.79 (0.39,1.59) | 0.507 |  |
| 2 | 18 | 0.00 (0.00,inf) | 0.998 |  |

Note: CI = confidence interval; OR = odds ratio; ^a^Adjusted for women’s age at embryo transfer.

**Supplemental Table 4** **Effect of HCG administrated in AC-FET cycles on early pregnancy loss rate in each subgroup.**

|  | No.of patients | OR (95%CI)^a^ | *P* value | *P* for interaction |
| --- | --- | --- | --- | --- |
| FET protocol |  |  |  | 0.058 |
| AC | 132 | 2.47 (0.95,6.38) | 0.063 |  |
| GnRH-a+AC | 54 | 0.21 (0.01,2.97) | 0.246 |  |
| Type of embryo transferred |  |  |  | 0.751 |
| Cleavage stage | 27 | 2.59 (0.11,60.31) | 0.553 |  |
| Blastocyst stage | 159 | 1.49 (0.63,3.50) | 0.365 |  |
| Number of embryos transferred |  |  |  | 0.360 |
| 1 | 147 | 2.05 (0.85,4.98) | 0.111 |  |
| 2 | 39 | 3.99 (0.21,76.49) | 0.359 |  |
| Number of good quality embryos transferred |  |  |  | 0.812 |
| 0 | 40 | 2.00(0.24,16.64) | 0.523 |  |
| 1 | 128 | 1.53 (0.56,4.24) | 0.410 |  |
| 2 | 18 | inf (0.00,inf) | 0.999 |  |

Note: CI = confidence interval; OR = odds ratio; ^a^Adjusted for women’s age at embryo transfer.
